# Association between the Anatomical Location of Glioblastoma and Its Evaluation with Clinical Considerations: A Systematic Review and Meta-Analysis

**DOI:** 10.3390/jcm13123460

**Published:** 2024-06-13

**Authors:** Juan Jose Valenzuela-Fuenzalida, Laura Moyano-Valarezo, Vicente Silva-Bravo, Daniel Milos-Brandenberg, Mathias Orellana-Donoso, Pablo Nova-Baeza, Alejandra Suazo-Santibáñez, Macarena Rodríguez-Luengo, Gustavo Oyanedel-Amaro, Juan Sanchis-Gimeno, Héctor Gutiérrez Espinoza

**Affiliations:** 1 Departamento de Ciencias Química y Biológicas, Facultad de Ciencias de la Salud, Universidad Bernardo O’Higgins, Santiago 8320000, Chile; juan.kine.2015@gmail.com; 2 Departament de Morfología, Facultad de Medicina, Universidad Andrés Bello, Santiago 8370146, Chile; ldmoyanov@gmail.com (L.M.-V.); vicentesb2003@gmail.com (V.S.-B.); danielmilos.b@gmail.com (D.M.-B.); pablo.nova@usach.cl (P.N.-B.); macarena.rodriguez@unab.cl (M.R.-L.); 3 Escuela de Medicina, Facultad Ciencias de la Salud, Universidad del Alba, Santiago 8320000, Chile; 4 Escuela de Medicina, Universidad Finis Terrae, Santiago 7501015, Chile; miorellanadonoso@gmail.com; 5 Department of Morphological Sciences, Faculty of Medicine and Science, Universidad San Sebastián, Santiago 8420524, Chile; 6 Faculty of Health and Social Sciences, Universidad de las Américas, Santiago 7500975, Chile; alej.suazo@gmail.com; 7 Facultad de Ciencias de la Salud, Universidad Autónoma de Chile, Santiago 8910060, Chile; g.oyanedelamaro@gmail.com; 8 GIAVAL Research Group, Department of Anatomy and Human Embryology, Faculty of Medicine, University of Valencia, 46001 Valencia, Spain; juan.sanchis@uv.es; 9 One Health Research Group, Universidad de las Americas, Quito 170124, Ecuador

**Keywords:** glioblastoma, encephalic glioblastoma, brain lobe, clinical anatomy

## Abstract

**Background:** Glioblastoma is a primary malignant brain tumor; it is aggressive with a high degree of malignancy and unfavorable prognosis and is the most common type of malignant brain tumor. Glioblastomas can be located in the brain, cerebellum, brainstem, and spinal cord, originating from glial cells, particularly astrocytes. **Methods:** The databases MEDLINE, Scopus, Web of Science, Google Scholar, and CINAHL were researched up to January 2024. Two authors independently performed the search, study selection, and data extraction. Methodological quality was evaluated with an assurance tool for anatomical studies (AQUA). The statistical mean, standard deviation, and difference of means calculated with the Student’s *t*-test for presence between hemispheres and presence in the frontal and temporal lobes were analyzed. **Results:** A total of 123 studies met the established selection criteria, with a total of 6224 patients. In relation to the mean, GBM between hemispheres had a mean of 33.36 (SD 58.00) in the right hemisphere and a mean of 34.70 (SD 65.07) in the left hemisphere, due to the difference in averages between hemispheres. There were no statistically significant differences, *p* = 0.35. For the comparison between the presence of GBM in the frontal lobe and the temporal lobe, there was a mean in the frontal lobe of 23.23 (SD 40.03), while in the temporal lobe, the mean was 22.05 (SD 43.50), and for the difference in means between the frontal lobe and the temporal lobe, there was no statistically significant difference for the presence of GBM, *p* = 0.178. **Conclusions**: We believe that before a treatment, it will always be correct to know where the GBM is located and how it behaves clinically, in order to generate correct conservative or surgical treatment guidelines for each patient. We believe that more detailed studies are also needed to show why GBM is associated more with some regions than others, despite the brain structure being homologous to other regions in which GMB occurs less frequently, which is why knowing its predominant presence in brain regions is very important.

## 1. Introduction

Glioblastoma is a primary malignant brain tumor. It is aggressive with a high degree of malignancy and unfavorable prognosis, and it is also the most common type of malignant brain tumor. This tumor of the central nervous system can be in the brain, cerebellum, brainstem, or spinal cord. It originates from glial cells, specifically the astrocyte. Based on their histological characteristics, astrocytomas are classified into grades ranging from I to IV. Grade IV corresponds to glioblastomas multiforme (GBM), which is the most unfavorable grade with a mean survival of 12–16 months even with treatment. Even with great advances in the pathogenesis and molecular biology of this tumor, it continues to have a poor prognosis once diagnosed.

According to the literature, the prevalence of GBM ranges from 0.59 to 5 per 100,000 inhabitants, being slightly higher for males and increasing in older patients. This disease is increasing in several countries, which may be attributed to various factors such as aging populations, air pollution, and technological advances associated with the use of more precise neurological examinations. However, the latter may lead to overdiagnosis of this pathology [1,2,3,4,5].

Depending on the mutation in the enzyme isocitrate dehydrogenase (IDH), a mutation that consists of a state of hypermethylation, astrocytoma can continue with a less aggressive or more aggressive clinical course, the latter being associated with wild-type IDH and linked to a primary tumor. Its progression has been described, with the presence of astrocyte-like stem cells in the astrocytic ribbon found in the frontal and temporal lobes close to the subventricular zone [4,6,7,8,9].

The objective of this review was to assess the location of GBM in the brain in terms of lobes, area, and hemisphere, and how it is related to specific clinical behaviors according to its location.

## 2. Methods

### 2.1. Protocol and Registration

This systematic review and meta-analysis were performed and reported according to the Preferred Reporting Items for Systematic Reviews and Meta-Analyses (PRISMA) statement [9]. The registration number in the International Prospective Register of Systematic Reviews (PROSPERO) is CRD42022224066.

### 2.2. Eligibility Criteria

Studies on the presence of GBM in different encephalic regions and its association with any clinical condition were considered eligible for inclusion if the following criteria were fulfilled: (1) population: sample of dissections or images of the presence of GBM; (2) outcomes: GBM presence, variants, and their correlation with symptomatology of the brain and other encephalic structures. Additionally, anatomical variants were classified and described based on normal anatomy and classifications and description proposed in the literature; (3) studies: this systematic review included research articles, research reports, and original research published in English in peer-reviewed journals and indexed in any of the databases reviewed. Conversely, the exclusion criteria were as follows: (1) population: animal studies; (2) studies that performed analysis of other involucred structures and pathologies outside the encephalic region; (3) studies: letters to the editor or comments.

### 2.3. Electronic Search

We systematically searched MEDLINE (via PubMed), Web of Science, Google Scholar, the Cumulative Index to Nursing and Allied Health Literature (CINAHL), and Scopus, from inception until January 2024. The search strategy included a combination of the following terms: “glioblastoma” (mesh), “encephalic glioblastoma” (no mesh), “brain lobe” (no mesh), and “clinical anatomy” (no mesh) using the Boolean connectors AND, OR, and NOT. The search strategies for each database are available in the Table 1.

### 2.4. Study Selection

Two authors (LM and VS) independently screened the titles and abstracts of references retrieved from the searches. We obtained the full text for references that either author considered to be potentially relevant. We involved a third reviewer (DM) if consensus could not be reached.

### 2.5. Data Collection Process

Two authors (JS and MO) independently extracted data on the outcomes of each study. The following data were extracted from the original reports: (i) authors and year of publication, (ii) type of study and subject numbers, (iii) incidence and anatomical location of GBM, (iv) statistical data, (v) geographical location, (vi) laterality, (vii) gender, (viii) relevant clinical considerations.

### 2.6. Assessment of the Methodological Quality of the Included Studies

The quality assessment was performed using the methodological quality assurance tool for anatomical studies (AQUA) proposed by the International Evidence-Based Anatomy Working Group (IEBA) [131,132,133]. Data extraction and quality assessment were independently performed by two reviewers (JJV and PN). We involved a third reviewer (JSG) if consensus could not be reached. The agreement rate between the reviewers was calculated using kappa statistics.

### 2.7. Statistical Methods

Regarding the statistical analysis, we calculated the mean and standard deviation of the subjects with GBM, grouped according to hemisphere and lobes. Then we collected the data and entered them into Excel software (v.12.1.3) for better tabulation. Statistical analysis was subsequently performed using the Student’s *t*-test to evaluate whether the population was normally distributed. This method is suitable for small groups, as in our research, and was carried out to assess the predominance of hemispheres and the predominance between the appearance of GBM in the frontal lobe and in the temporal lobe.

## 3. Results

**Figure 1:**
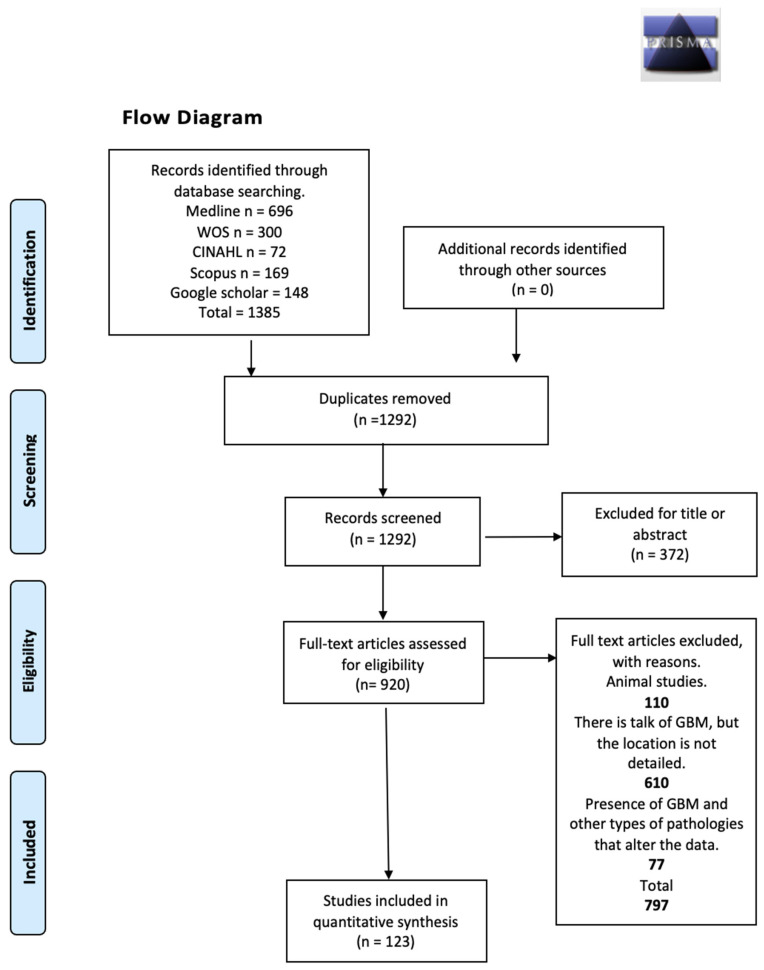
Search diagram.

In this review, 121 studies were included with a sample size of 6224 patients with glioblastoma, of which 64 studies corresponding to 52.89% of the total were retrospective studies [10,11,12,13,14,15,16,17,18,19,20,21,22,23,24,25,26,27,28,29,30,31,32,33,34,35,36,37,38,39,40,41,42,43,44,45,46,47,48,49,50,51,52,53,54,55,56,57,58,59,60,61,62,63,64,65,66,67,68,69,70,71,72,73], 27 (22.31%) prospective studies [74,75,76,77,78,79,80,81,82,83,84,85,86,87,88,89,90,91,92,93,94,95,96,97,98,99,100], 24 (19.83%) case reports [101,102,103,104,105,106,107,108,109,110,111,112,113,114,115,116,117,118,119,120,121,122,123,124], 3 (2.48%) cadaveric studies [125,126,127], 2 (1.65%) cross-sectional studies [128,129], and finally, only 1 (0.83%) study that presented a case report along with a literature review [130]. The samples included in the studies were distributed across all continents with the exception of Africa; the Asian continent was represented by a total of 56 studies corresponding to 46.28% of the total studies and a sample of 2801 corresponding to 45.00% of the total patients [10,12,16,17,19,20,21,24,27,30,31,32,35,36,39,40,41,42,43,44,46,49,51,53,59,65,66,67,68,69,71,73,75,76,77,83,86,87,88,91,93,94,97,99,101,103,105,107,109,111,116,119,120,122,124], the European continent accounted for a total of 34 (28.10%) studies and 1281 (20.58%) patients [15,18,28,29,45,47,48,50,57,61,63,70,72,74,78,79,80,81,82,84,85,89,90,95,96,102,104,112,113,117,123,125,130]; North America contributed 28 (23.14%) studies and 2030 (32.62%) patients [11,13,22,23,33,34,37,52,54,55,56,58,60,64,87,92,98,100,106,108,110,114,115,118,126,127,128,129]; and South America provided only 3 (2.48%) studies and a total of 112 (1.89%) patients [14,38,121]. With respect to sex, 104 studies detailed the sex of patients with glioblastoma and 17 studies only mentioned the total number of patients without differentiating them by sex and/or differentiating some only some or a few specific cases from the total presented [13,15,25,34,39,41,45,50,53,64,66,68,74,84,86,88,112]. Therefore, 1949 patients were counted for the female sex, corresponding to 31.31% of the total, and2873 patients were counted for the male sex, corresponding to 46.16% of the total. In the same way, the number of patients who were not differentiated by sex was counted, obtaining 1402 undetermined patients, corresponding to 22.53% of the total (Figure 1).

### 3.1. Statistical Results

Reviewing the articles consulted, a total of 1768 cases were reported, with a mean of 33.36 and a standard deviation of 58.00, for those who presented glioblastoma in the right hemisphere of the brain, while for the left hemisphere, there were 1664 cases, with a mean of 34.70 and a standard deviation of 65.07. It is worth mentioning that the table refers to certain cases where the number of patients diagnosed with glioblastoma did not coincide with the data on the number of hemispheres affected, due to the following possible reasons: the articles considered patients who did not present glioblastoma, they did not detail all patients with glioblastoma, laterality was not specified, or the classification was imprecise [13,15,17,26,32,37,64,66,74,83,125,130] (Table 2 and Table 3).

For the statistical values found between the differences in the presence of GBM in the right hemisphere (HR) and the left hemisphere (HL), we employed the Shapiro–Wilk test to examine the normal distribution of GBM presence in each hemisphere. The test gave a *p*-value of <0.001, indicating a statistically significant difference between the means of the samples included for the analysis. It is important to note that this refers only to the sample means and not to the presence of GBM itself. For the statistical difference of means between the presence of GBM between hemispheres, the scores for the HR were a mean of 35.5 and a SD of 68.9, which were higher than those for HL, which were a mean of 22.6 and a SD of 37.0. Although the presence of GBM in the HR was higher, the Student’s *t*-test showed that there was no statistically significant difference for the presence of GBM in the interhemispheric comparison *p* = 0.352.

**Figure 2:**
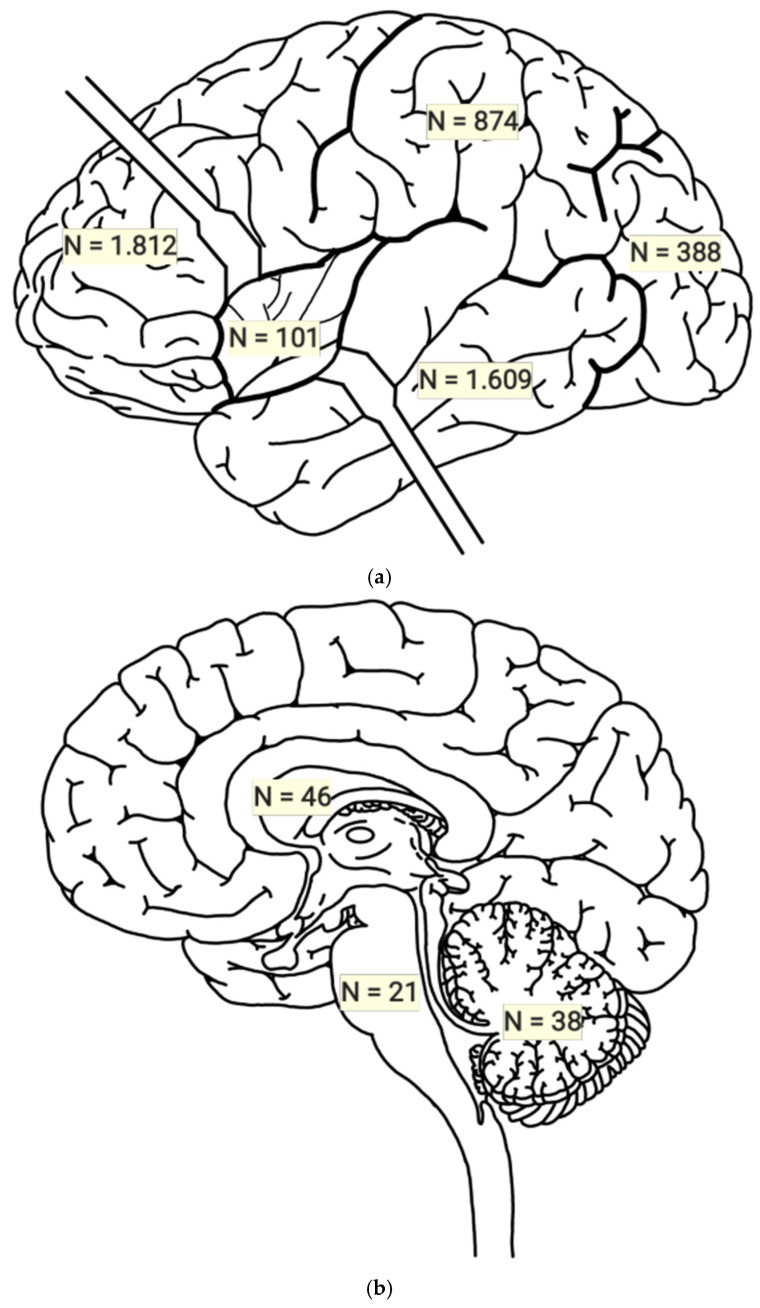
(**a**,**b**): Numbers of subjects with glioblastoma per brain lobe.

**Figure 3:**
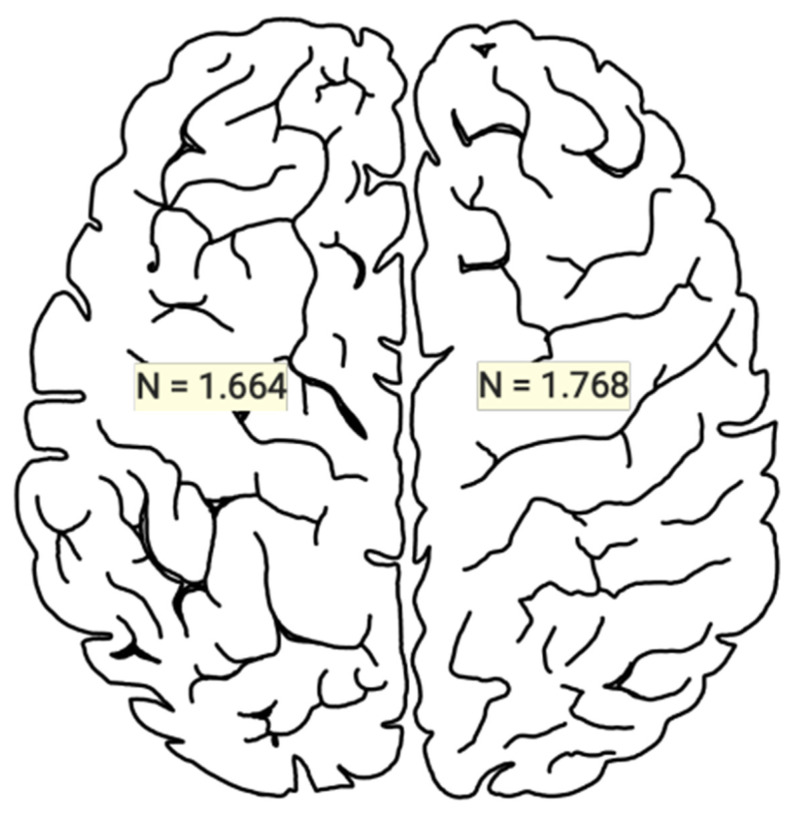
Numbers of subjects with glioblastoma per cerebral hemisphere.

The total number of cases reported as affected for the following brain regions were as follows: frontal lobe in 1812 cases, with a mean of 23.23 and a standard deviation of 40.03; parietal lobe in 874 cases, with a mean of 14.81 and a standard deviation of 28.56; temporal lobe in 1609 cases, with a mean of 22.04 and a standard deviation of 43.50; occipital lobe in 388 cases, with a mean of 8.62 and a standard deviation of 17.52; insula in 101 cases, with a mean of 9.18 and a standard deviation of 14.86; diencephalon in 46 cases, with a mean of 3.06 and a standard deviation of 2.69; brainstem in 21 cases, with a mean of 1.62 and a standard deviation of 1.12; cerebellum in 38 cases, with a mean of 2.71 and a standard deviation of 2.58; and other structures in 1275 cases, with a mean of 21.61 and a standard deviation of 37.25. However, differences were observed between the number of patients diagnosed with glioblastoma and the number of regions affected by the tumor, which may be due to any of the following reasons: locations of patients with different types of gliomas were reported without making a difference; of those who presented glioblastoma, it was detailed or assumed that patients manifested more than one affected area; or not all of the regions involved were reported [13,15,17,26,32,37,64,66,74,83,125,130] (Table 4) and Figure 2 and Figure 3.

Regarding the statistical values found between the differences in the presence of GBM in different lobes, we considered only the frontal lobe and the temporal lobe for the analysis since in our study, these two were the lobes with the highest proportions of cases. It was more correct to analyze them according to the number of appearances in the population of each study. In relation to the above, for the frontal lobe and the temporal lobe, we used the data to calculate the normal distribution between the presence of GBM in the frontal lobe and GBM in the parietal lobe. The result was <0.180, showing that there was no statistically significant difference between the means of the samples included for the analysis. For the statistical difference of means between the presence of GBM between frontal and parietal lobes, where the values obtained for the frontal lobe were a mean of 23.0 and an SD of 23.3, which were lower than those of the temporal lobe, which were a mean of 27.5 and an SD of 24.8, although the presence of GBM in the temporal lobe was greater, the Student’s *t*-test result of 0.178 showed no statistically significant difference for the presence of GBM between lobes.

### 3.2. Risk of Bias of Included Studies

**Figure 4:**
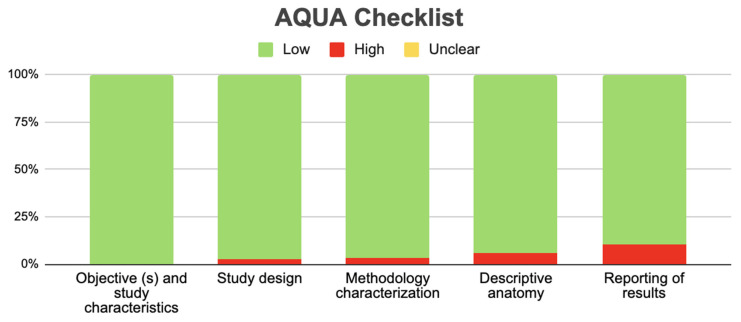
AQUA Checklist.

In total, 123 articles were evaluated with the AQUA Checklist to analyze the risk of bias in five domains. For the first domain, which covers the description of the objectives and characteristics of the study, all studies presented a low risk of bias. The second domain is the correct reporting of the study design; 120 studies presented a low risk of bias in this domain, and 3 presented a high risk since they did not clearly report the design of their studies [12,29,42]. For the third domain, which analyzes the study’s methodological characteristics, 119 studies presented a low risk of bias, while 4 presented a high risk since their methodology was unclear [29,39,67,69]. The fourth domain is the correct description of anatomy; 116 studies presented a low risk of bias in this domain, while 7 studies presented a higher risk since they did not include an anatomical description of the variant but instead merely named it [15,19,31,37,47,69,73]. In the final domain, which involves reporting results, 110 studies presented a low risk of bias, and 13 studies presented a high risk of bias since their results were presented diffusely in tables or in discussion sections [17,20,22,23,24,37,40,52,54,58,69,71,75] (Figure 4).

### 3.3. Clinical Implications

The clinical implications associated with the anatomical location of glioblastoma were analyzed in different articles, with a total of 107 articles in which a relationship was not made between the region of glioblastoma and a clinical implication [10,11,13,18,19,20,21,22,24,25,26,27,28,29,30,31,32,33,34,35,36,37,38,39,40,41,42,43,44,45,46,47,48,49,50,51,52,53,54,55,56,57,58,59,60,61,62,63,64,65,66,67,68,70,71,72,73,74,75,77,78,79,80,81,82,83,84,85,87,88,89,90,91,92,93,94,95,96,97,98,99,100,104,105,107,108,109,110,111,112,113,114,115,116,117,118,119,120,121,123,124,125,126,127,128,129,130]. Regarding tumor location being considered an important factor, it has been suggested that depending on the specific area affected, the tumor’s aggression and its impact on patient survival can be understood. Furthermore, it is considered that the most frequently involved areas are the frontal and temporal lobes, both of which have different clinical properties. In the review of the articles, it was found that among patients diagnosed with glioblastoma, those who were advanced in age had a greater likelihood of the affected area(s) being the bilateral temporal lobe, while in young patients, the tumor was predominantly located in the left lower frontal region [14,23,42,69].

The possible locations of glioblastoma include the infratentorial region. These tumors are considered unusual, complex, and rare, due to the low life expectancy of patients who present them. In adults, the incidence is in the range of 1.2% of all patients with glioblastoma, however, in infants, the infratentorial location is predominant. This type of glioblastoma can be difficult to diagnose and even misleading due to nonspecific symptoms and radiological characteristics. Nevertheless, a common clinical characteristic described is a rapid deterioration of ataxia and dysmetria, and likewise, compression of segments of the spinal cord can lead to lower extremity movement disorders, pain, and abnormal urination. It is worth mentioning that based on what was described by Stark [15], it is considered that from a pathological point of view, infratentorial glioblastoma has similar behavior to the supratentorial type [15,16,17,101,103].

Regarding the symptoms and signs reported in the review, an apparent relationship can be established between glioblastoma located in the frontal lobe and mood symptoms; however, it is considered not significant. On the other hand, it has been described that the spinal metastatic dissemination of intracranial glioblastoma, which is usually simultaneous or sequential to the progression of the latter, does not present symptoms in most patients or these are of late onset. Moreover, these symptoms are often masked by intracranial progression of the tumor, which leads to motor disorders and sensory anomalies. Another observation made is regarding the location in the pineal region, which in addition to being considered very rare and with an unfavorable prognosis, is associated with a high frequency of hydrocephalus and Parinaud syndrome [17,102,106].

Finally, when comparing glioblastoma to other pathologies, primary brain lymphoma is mentioned, presenting important differences in terms of the anatomical location of the tumor. Regarding solitary brain metastases, there appears to be no difference between sex. However, patients with solitary brain metastases are older than those with glioblastoma [12,16,76,86].

## 4. Discussion

This systematic review and meta-analysis aimed to explore the different locations of GBM and their association with various clinical considerations. For this purpose, a review of different studies related to the presence of glioblastoma was conducted, covering topics such as anatomical location, descriptions, characteristics, and prevalence within the different areas of the brain and spinal cord. Based on our inclusion and exclusion criteria, a total of 121 articles were identified. The main finding of this review is that GBM can occur in various regions of the brain and depending on the location, it may present with varied symptomatology leading to diverse differential diagnoses.

In relation to previous review studies that have analyzed the anatomical location of glioblastoma, we did not find any that report the same relationship as presented in this meta-analysis. Therefore, this study represents a first-time effort in filtering and analyzing information on this manner, making it a novel and up-to-date study. However, when searching the databases with the search string “brain” OR “encephalic” location of glioblastoma, we found three studies that mentioned some type of information in their sections.

The study by Zarnet [134], which mentions among its results that one of the main locations of GBM is the premotor cortex but focuses mainly on treatment through radiotherapy, did not make a detailed study of the anatomy and location of the GBM, thus presenting very low similarity to our review. The study by Maslehaty [130], which showed as its main results that GBMs could be located not only in specific encephalic structures but also in leptomeninges, without specifying which of these meninges, and also proposed radiotherapy as the gold standard for possible treatment. Again, our review differs due to the characteristics previously mentioned. Lastly, the review by Corr [135] showed as its main results that there has been a lack of prospective studies analyzing the prognostic characteristics of glioblastoma together with its anatomical and radiological characteristics, which would eventually facilitate the early identification of glioblastoma recurrence, thus supporting a more personalized treatment and follow-up strategy. Although that study does not make the case specifically, it supports the findings of the current review and highlights the importance of specific knowledge and location of the GBM which can lead to better management and treatment of patients with this pathology.

The characteristics of the included studies were as follows. For this review, after applying our inclusion and exclusion criteria, 121 were included with a total sample of 6224 subjects with GBM. Regarding the geographical location, the distribution was primarily across the continents of Asia, Europe, and North America. Therefore, it cannot be inferred that the presence of GBM is associated with any geographic region or that it is linked to any ethnicity or race. Moreover, the gender distribution showed a higher occurrence in men than in women; however, the difference was not statistically significant, indicating that the presence of GBM cannot be associated with a specific sex. We believe all these characteristics should be validated and supported by further studies; although the sample of this review is representative, new studies could confirm or refute these exposed results. One of the strengths we believe this review contributes is the statistical report we present regarding the characteristics and location of GBM. Concerning the hemisphere where GBM was found, there were no differences between the left and right hemispheres of the brain. In the right hemisphere, the mean was 33.36 subjects, while for the left hemisphere, it was 34.7. Although the mean occurrence of interhemispheric GBM presence was higher in the right hemisphere, the Student’s *t*-test for GBM presence being greater in one hemisphere than the other showed no statistically significant difference, leading us to believe that there is no predisposition for the appearance of GBM in a hemisphere, independent of the subject’s motor dominance. Thus, our results reject the supposition that the appearance is associated with motor predominance. Regarding the location of GBM in relation to lobes, the main lobes where GBM was located were the frontal and temporal lobes.

Therefore, we performed statistical calculations comparing only these two lobes, understanding that if we compared them with other lobes, these two would always show a statistically higher presence of GBM. The comparison between the presence of GBM in the frontal and temporal lobes showed no statistically significant differences between the frontal and temporal lobe samples. In the comparison between its presence in one lobe over the other, GBM was more frequent in the temporal lobe, but the difference in its presence in this lobe compared with the frontal lobe was not statistically significant. Therefore, these data indicate that, while mainly located in these lobes, there was no result stating that GBM was specifically located more in one lobe than in the other. In the bias analysis of the included studies, the majority presented a moderate and low risk of bias, meaning the results can be correctly interpreted. However, caution is always advised since there were some domains that were not correctly met, which could alter the primary reported results to some extent.

Among our main objectives was to find evidence on the relationship between the region affected by glioblastoma and the clinical considerations it may entail. For this purpose, of the total sources consulted, 20 were used. However, we consider that the studies most relevant in support of our purpose were those that described the following: ataxia and dysmetria for infratentorial glioblastomas; the relationship between spinal segment disorders and disorders of lower limb movement, pain, and abnormal urination; a non-significant relationship between tumors in the frontal lobe and mood alteration; movement disorders and sensory anomalies with the progression of intracranial glioblastoma; and finally, hydrocephalus and Parinaud syndrome with glioblastoma in the pineal region.

In the analysis of the clinical considerations associated with infratentorial glioblastomas, such as those located in the cerebellum or spinal cord, we believe that the effects presented by patients with these conditions are due to alterations in the pathways that conduct sensorimotor information preventing, for example, adequate coordination of the lower extremities when walking, or simply their individual movement. Likewise, depending on the spinal segment that is affected by the glioblastoma, essentially the lumbar and/or sacral region, it could influence the innervation of the urinary system and, therefore, normal urination. We believe that intracranial progression can lead to such alterations because the brain regions responsible for developing and coordinating movements may be affected, along with areas for integration of sensory information. On the other hand, given the limited information known about the functioning of the pineal gland, we are unsure of its relationship with the conditions it generates when a tumor and/or glioblastoma develops in that location. Finally, regarding the relationship between the frontal lobe and mood, we consider that it should continue to be studied because, although the literature mentions that there is a relationship, it has been described as non-significant and, furthermore, no reference has been made to the limbic system, the main structure associated with the emotional state.

Although an assumption can be made about the explanation of the relationship between the anatomical region affected by glioblastoma and its clinical implications, we consider that it is important that future studies give it greater focus, to better understand the signs and symptoms that patients diagnosed with the pathology present or may present, with the aim of being able to provide adequate care and treatment and, at the same time, expanding knowledge in the area.

## 5. Limitations

This review was limited by the publication and authorship bias of the included studies. Firstly, studies with different results that were in non-indexed literature in the selected databases may have been excluded. Secondly, there may have been limitations in the sensitivity and specificity of the searches. Finally, the authors personally selected articles. All of this increases the probability of excluding potential cases from countries outside of Asia and North America that have not been reported in the scientific community.

## 6. Conclusions

The presence of a GBM will always be detrimental to the correct functioning of the brain structures. Knowledge of the specific location and area of the cortex that is affected by the GBM can teach us to better understand the clinical implications and avoid some types of differential diagnoses. This knowledge can help develop appropriate conservative or surgical treatment strategies for each patient. Future studies that can address the behavior of glial cells and the types of conditions that could contribute to the formation of GBM, with investigation of why this occurs more in some regions than in others, may be crucial.

## Figures and Tables

**Table 1:** Characteristics of the included studies.

Author	Study Type and Number of Subjects.	Incidence and Anatomical Location of GB	Relevant Statistical Data	Geographic Location	Laterality	Sex of Patients with GBM	Relevant Clinical Considerations
Hashiguchi 2022 [10]	Retrospective study, 51 patients.	51/51 (100%) Frontal: 15 Temporal: 16 Parietal: 8 Occipital: 3 Cerebellum: 2 Others (including midline lesion or multiple lesions): 7	Not specified	Japan	Laterality not reported.	25 female; 26 male.	The article did not establish a relationship between the region of the glioblastoma and clinical implications.
Drabycz 2009 [11]	Retrospective study, 72 patients	72/72 (100%) Frontal: 19 Temporal: 32 Parietal: 17 Occipital: 2 Declassified: 2	*p*-value: 0.85 in relation to the grouping of the right and left sides or predominant lobe; *p*-value: 0.88 in relation to the comparison of the occupation of the left and right sides.	Canada	R. hemisphere: 34 L. hemisphere: 38	24 female; 48 male.	There was no association between the anatomical location or radial distribution of GBM and the MGMT promoter methylation status.
Ko 2016 [12]	Retrospective study, 126 patients	104/126 (82.5%) Frontal: 38 Parietal: 25 Temporal: 27 Occipital: 3 Basal ganglia: 2 Corpus callosum: 3 Thalamus: 5 Brainstem: 1.	*p*-value: 0.02 in relation to the location of the tumor.	Taiwan	Laterality not reported	46 female; 58 male.	There were statistically significant differences in tumor location between GBM and primary brain lymphoma.
Abecassis 2020 [13]	Retrospective study, 100 patients	31/100 (31%) Frontal: 17 Parietal: 8 Temporal: 24 Occipital: 0 Brainstem: 1 Cerebellum: 0 These data correspond to the locations of tumors from patients with gliomas of different grades, not specifically GBM.	*p*-value: < 0.001 in relation to tumor location of patients with glioma, not specifically GBM.	USA	R. hemisphere: 20 L. hemisphere: 30; for patients with gliomas of different grades.	Not specified	The article did not establish a relationship between the region of the glioblastoma and clinical implications.
Ferreira 2004 [14]	Retrospective study, 67 patients	67/67 100% Frontal: 32 (2 bilateral) Temporal: 12 Parietal: 11 Occipital: 4 (1 bilateral) Frontal and temporal: 1 Temporo-parietal: 1 Parieto-occipital: 1 Parieto-occipital + Corpus callosum: 1 Corpus callosum: 3 Brainstem: 1	Not specified	Brazil	Laterality not reported	29 female; 38 male.	Regarding their location, they were more frequent in the frontal and temporal lobes. In children, the most frequent location was infratentorial.
Stark 2010 [15]	Retrospective study, 7 patients	7/7 (100%) Cerebellum: 4 Brainstem: 2 Inferior to the floor of the fourth ventricle: 1	Not specified	Germany	L. hemisphere: 1; lateralities of the remaining cases were not reported.	1 female; 1 male. Gender was not reported for the remaining cases, so it was considered as NS.	The incidence of infratentorial glioblastomas (iGB) in adults is in the range of 1.2% of all GB patients. The most common clinical features of iGB are rapid deterioration of ataxia and dysmetria. Diagnosing iGB can be challenging due to nonspecific symptoms and radiological characteristics, leading to misdiagnosis. The pathological findings of iGB are comparable to supratentorial glioblastomas.
Liu 2018 [16]	Retrospective study, 167 patients	107/167 (64.07%) Basal ganglia and thalamus: 4 Cortex: 68 Subventricular zone: 64 Corpus callosum: 17 several patients presented with more than one location.	*p*-value: <0.001 in relation to location in the basal ganglia and thalamus and cortex; *p*-value: 0.076 in relation to location in the subventricular zone; *p*-value: 1.000 in relation to location in the corpus callosum.	China	Laterality not reported	34 female; 73 male.	GBMs located in infratentorial regions or multiple lesions are rare. There are significant differences in the anatomical location between GBM and primary central nervous system lymphoma.
Chen 2022 [17]	Retrospective study, 15 patients	15/15 (100%) Frontal: 5 Temporal: 3 Occipital: 1 Temporo-occipital: 1 Parieto-occipital: 1 Corpus callosum: 1 Cerebellum: 1 Brainstem: 1 Spinal (C1–C7): 1	Not specified	China	R. hemisphere: 4 L. hemisphere: 9	6 female; 9 male.	Compression of the affected segments of the spinal cord results in dysfunction, often presenting as lower limb movement disorders, pain, and abnormal urination. As spinal metastatic dissemination usually occurs simultaneously or sequentially with the progression of intracranial GBM, patients are often asymptomatic or of late onset. Progression of intracranial GBM leads to motor disorders and sensory abnormalities, which may additionally mask symptoms of spinal metastatic spread.
Jaskólski 2013 [18]	Retrospective study, 89 patients	26/89 (29.2%) Frontal: 11 Temporal: 7 Parietal: 6 Occipital: 2	Not specified	Poland	Laterality not reported	12 female; 14 male.	The article did not establish a relationship between the region of the glioblastoma and clinical implications.
Kim 2019 [19]	Retrospective study, 83 patients	83/83 100% Frontal or temporal: 29 Others: 54	*p*-value: 0.19 in relation to the tumor location.	South Korea	Laterality not reported	37 female; 46 male.	The article did not establish a relationship between the region of the glioblastoma and clinical implications.
Hatakeyama 2021 [20]	Retrospective study, 75 patients	55/75 (73.3%) Cerebral hemisphere: 49 Supratentorial central structures: 5 Vermis: 1	Not specified	Japan	Laterality not reported	22 female; 33 male.	The article did not establish a relationship between the region of the glioblastoma and clinical implications.
Yamashita 2019 [21]	Retrospective study, 112 patients	112/112 (100%) Frontal: 36 Parietal: 25 Temporal: 21 Occipital: 3 Insula: 7 Basal ganglia or thalamus: 16 Brainstem or cerebellum: 4	Not specified	Japan	Laterality not reported	56 female; 56 male.	The article did not establish a relationship between the region of the glioblastoma and clinical implications.
Kuroiwa 1995 [22]	Retrospective study, 9 patients	9/9 (100%) Cerebellar vermis: 3 Cerebellar vermis and brainstem: 2 Cerebellar hemisphere: 1 Vermis and cerebellar hemisphere: 1 Cerebellar hemisphere and brainstem: 1 Brainstem: 1.	Not specified	USA	Laterality not reported	1 female; 8 male.	The article did not establish a relationship between the region of the glioblastoma and clinical implications.
Awad 2017 [23]	Retrospective study, 330 patients	330/330 (100%) Frontal: 132 Temporal:139 Parietal: 99 Occipital: 50 Periventricular: 11 Hippocampal: 51 Brainstem: 4 Basal ganglia/deep nuclei: 32 Cerebellum: 3	Univariate *p*-value: 0.32 in relation to frontal location; 0.918 in relation to temporal location; 0.336 in relation to parietal location; 0.121 in relation to occipital location; 0.006 in relation to periventricular location; 0.304 in relation to hippocampal location; 0.114 in relation to brainstem location; 0.002 in relation to deep nuclei/basal ganglia location; 0.852 in relation to cerebellar location. Multivariate *p*-value: 0.816 in relation to occipital location; 0.518 in relation to periventricular location; 0.28 in relation to brainstem location; 0.045 in relation to deep nuclei/basal ganglia location.	USA	R. hemisphere: 168 L. hemisphere: 139 Bilateral: 23	130 female; 200 male.	It was suggested that specific tumor locations may play a significant role in better understanding the aggressive nature of GBM and how it impacts patient survival.
Quan 2023 [24]	Retrospective study, 110 patients	110/110 (100%) Frontal: 42 Others: 68	*p*-value: 0.204 in relation to the location, or not, of the tumor in the frontal lobe.	China	Laterality not reported	49 female; 61 male.	The article did not establish a relationship between the region of the glioblastoma and clinical implications.
Onuma 2013 [25]	Retrospective study, 33 patients	33/33 (100%) Frontal: 17 Others: 16	*p*-value: 0.157 in relation to the location, or not, of the tumor in the frontal lobe.	Japan	Laterality not reported	Not specified	The article did not establish a relationship between the region of the glioblastoma and clinical implications.
Cui 2021 [26]	Retrospective study, 77 patients	77/77 (100%) Frontal: 44 Frontal/temporal insula lobe: 22 Parietal/parieto-occipital lobe: 11 All 77 had the corpus callosum affected.	*p*-value: 0.121 in relation to location in the frontal lobe; *p*-value: 0.571 in relation to location in the frontal/temporal insula lobe; *p*-value: 0.273 in relation to location in the parietal/parieto-occipital lobe.	China	Unilateral: 41 Blateral: 36; for unilateral cases, the hemisphere was not specified.	44 female; 43 male.	The article did not establish a relationship between the region of the glioblastoma and clinical implications.
Chen 2023 [27]	Retrospective study, 42 patients	20/42 (47.6%) Frontal: 12 Parietal: 3 Temporal: 2 Basal ganglia: 1 Cerebellar hemisphere: 2 The corpus callosum was invaded by 3 frontal lesions.	Not specified	China	Laterality not reported	8 female; 12 male.	The article did not establish a relationship between the region of the glioblastoma and clinical implications.
Wach 2020 [28]	Retrospective study, 198 patients	198/198 (100%) R. hemisphere: 111 L. hemisphere: 87	*p*-value: 0.398 in relation to the location in the right hemisphere.	Germany	R. hemisphere: 111 L. hemisphere: 87	80 female; 118 male.	The article did not establish a relationship between the region of the glioblastoma and clinical implications.
Steidl 2023 [29]	Retrospective study, 65 patients	65/65 (100%) Temporal: 27 Frontal: 16 Parietal: 15 Occipital: 12 Thalamus: 2 Insula: 1 Basal ganglia: 1	Not specified	Germany	R. hemisphere: 36 L. hemisphere: 28 Bilateral: 1	17 female; 48 male.	The article did not establish a relationship between the region of the glioblastoma and clinical implications.
Park 2017 [30]	Retrospective study, 108 patients	108/108 (100%) Frontal: 45 Others: 63	*p*-value: 0.955 in relation to the location of GBM in other areas.	South Korea	Laterality not reported	54 female; 54 male.	The article did not establish a relationship between the region of the glioblastoma and clinical implications.
Ideguchi 2015 [31]	Retrospective study, 5 patients	5/5 (100%) Frontal: 1 Temporal: 1 Parietal: 1 Occipital: 1 Basal ganglia: 1	Not specified	Japan	R. hemisphere: 3 L. hemisphere: 2	4 female; 1 male.	The article did not establish a relationship between the region of the glioblastoma and clinical implications.
Wang 2018 [32]	Retrospective study, 34 patients	19/34 (55.9%) Frontal: 7 (1 bilateral) Temporal: 1 Parietal: 2 Frontal, occipital, temporal: 1 Fronto-temporal: 1 Corpus callosum: 1 Brainstem: 1 Basal ganglia: 1 Not specified: 4	Not specified	China	R. hemisphere: 6 L. hemisphere: 6 Bilateral: 1. Not specified: 6.	4 female; 15 male.	The article did not establish a relationship between the region of the glioblastoma and clinical implications.
Muller 2019 [33]	Retrospective study, 275 patients	275/275 (100%) R. hemisphere: 141 L. hemisphere: 134	Not specified	USA	R. hemisphere: 141 L. hemisphere: 134	105 female; 170 male.	The article did not establish a relationship between the region of the glioblastoma and clinical implications.
Thomas 2016 [34]	Retrospective study, 21 patients	21/21 (100%) Corpus callosum both hemispheres (butterfly): 5 Insula: 4 Thalamus/basal ganglia: 1 Cingulate: 2 Splenium: 2 Temporal: 1 Motor area: 3 Speech area (broca): 3	Not specified	USA	Laterality not reported	Not specified	The article did not establish a relationship between the region of the glioblastoma and clinical implications.
Koike 2022 [35]	Retrospective study, 22 patients	11/22 (50%) Infratentorial: 11 Supratentorial: 0	*p*-value: 0.279	Japan	Laterality not reported	5 female; 6 male.	The article did not establish a relationship between the region of the glioblastoma and clinical implications.
Xing 2018 [36]	Retrospective study, 75 patients	75/75 (100%) Frontal: 32 Parietal: 12 Temporal: 13 Occipital: 5 Insula: 1 Others: 12	*p*-value: 0.002	China	Laterality not reported	34 female; 41 male.	The article did not establish a relationship between the region of the glioblastoma and clinical implications.
Smedley 2018 [37]	Retrospective study, 304 patients	304/304 (100%) Frontal: 114 Temporal: 91 Parietal: 76 Occipital: 18 Thalamus: 8 Corpus callosum: 4 Cerebellum: 1 Pineal gland: 1 Midbrain: 1 In 7 cases it was bilateral and in 11 cases it involved 2 locations.	Not specified	USA	R. hemisphere: 162 L. hemisphere: 149	116 female; 188 male.	The article did not establish a relationship between the region of the glioblastoma and clinical implications.
Miquelini 2016 [38]	Retrospective study, 84 patients	42/84 (50%) Supratentorial: 41 Infratentorial: 1 Three cases presented involvement of the corpus callosum	*p*-value: 0.007 in relation to infratentorial location; *p*-value: 0.048 in relation to the cases involving the corpus callosum.	Argentina	Laterality not reported	19 female; 23 male.	The article did not establish a relationship between the region of the glioblastoma and clinical
Han 2018 [39]	Retrospective study, 92 patients	92/92 (100%) R. hemisphere: 39 L. hemisphere: 39 Midline: 14.	*p*-value: 0.72 in relation to presence in the left hemisphere.	China	R. hemisphere: 39 L. hemisphere: 39 Midline: 14.	Not specified	The article did not establish a relationship between the region of the glioblastoma and clinical implications.
Mathew 2018 [40]	Retrospective study, 47 patients	47/47 (100%) Ipsilateral cerebral lobes: 15 Lateral ventricle: 41	Not specified	India	Laterality not reported	14 female; 33 male.	The article did not establish a relationship between the region of the glioblastoma and clinical implications.
Jiang 2017 [41]	Retrospective study, 10 patients	10/10 (100%) Single lobe: 7 Parieto-occipital: 1 Third ventricle: 2	Not specified	China	Laterality not reported	Not specified	The article did not establish a relationship between the region of the glioblastoma and clinical implications.
Li 2018 [42]	Retrospective study, 406 patients	406/406 (100%) Frontal: 182 Temporal: 224	*p*-value: 0.879 in relation to the hemispheres.	China	R. hemisphere: 179 L. hemisphere: 227	195 female; 211 male. Female: 77 frontal lobe and 118 temporal lobe; male: 105 frontal lobe and 106 temporal lobe.	The results demonstrated that tumor location was an important factor, and glioblastomas in the frontal lobe and temporal lobe had different clinical properties.
Utsuki 2005 [43]	Retrospective study, 37 patients	37/37 (100%) Frontal: 16 Temporal: 8 Parietal: 3 Occipital: 5 Parietoocipital: 4 Frontoparietal: 1	Not specified	Japan	Laterality not reported	18 female; 19 male.	The article did not establish a relationship between the region of the glioblastoma and clinical implications.
Fan 2017 [44]	Retrospective study, 133 patients	133/133 (100%) R. hemisphere: 56 L. hemisphere: 77	Not specified	China	R. hemisphere: 56 L. hemisphere: 77	51 female; 82 male.	The article did not establish a relationship between the region of the glioblastoma and clinical implications.
Hart 2016 [45]	Retrospective study, 5 patients	5/5 (100%) Parietal: 2 Occipital: 1 Paracentral: 1 Postcentral and supramarginal gyri: 1	Not specified	UK	R. hemisphere: 5	Not specified	The article did not establish a relationship between the region of the glioblastoma and clinical implications.
Wang 2014 [46]	Retrospective study, 153 patients	153/153 (100%) R. hemisphere: 73 L. hemisphere: 80	Not specified	China	R. hemisphere: 73 L. hemisphere: 80	56 female; 97 male.	The article did not establish a relationship between the region of the glioblastoma and clinical implications.
Smets 2013 [47]	Retrospective study, 24 patients	24/24 (100%) Frontal: 4 Fronto-parietal: 1 Occipital: 2 Parietal: 5 Parieto-occipital: 4 Temporal: 6 Temporo-parietal: 2	Not specified	Belgium	R. hemisphere: 15 L. hemisphere: 9	11 female; 13 male.	The article did not establish a relationship between the region of the glioblastoma and clinical implications.
Eoli 2007 [48]	Retrospective study, 86 patients	86/86 (100%) Frontal: 36 Temporal: 35 Others: 15	*p*-value: 0.005 in relation to the frontal lobe and temporal lobe.	Italy	Laterality not reported	21 female; 65 male.	The article did not establish a relationship between the region of the glioblastoma and clinical implications.
sugimoto 2015 [49]	Retrospective study, 4 patients	4/4 (100%) Temporal: 1 Frontal: 3 Case 3 presented a satellite lesion in the right frontal lobe via the corpus callosum.	Not specified	japan	R. hemisphere: 0 L. hemisphere: 3 Bilateral: 1	4 female	The article did not establish a relationship between the region of the glioblastoma and clinical implications.
seidel 2011 [50]	Retrospective study, 122 patients	122/122 (100%) Frontal: 70 Temporal: 62 Central: 62 Parieto-occipital: 38 Basal ganglia: 10 Others: 2	Not specified	Germany	Laterality not reported	Not specified	The article did not establish a relationship between the region of the glioblastoma and clinical implications.
Cho 2018 [51]	Retrospective study, 60 patients	60/60 (100%) Frontal: 33 Parietal: 21 Temporal: 34 Occipital: 4 Insula: 8 Deep gray matter: 14 Corpus callosum: 8 Midbrain: 5 Infratentorial: 2 several patients presented with more than one location.	Not specified	South Korea	Laterality not reported	25 female; 35 male.	The article did not establish a relationship between the region of the glioblastoma and clinical implications.
Olar 2012 [52]	Retrospective study, 9 patients	4/9 (44.44%) Temporal: 1 Parietal: 1 Fronto-temporo-parietal: 1 Basal ganglia and insula: 1	Not specified	USA	R. hemisphere: 1 L. hemisphere: 3	1 female; 3 male.	The article did not establish a relationship between the region of the glioblastoma and clinical implications.
woo 2019 [53]	Retrospective study, 147 patients	147/147 (100%) Temporal: 47 Frontal: 46 Parietal: 34 Occipital: 5 Insula: 3 Corpus callosum: 3 Cerebellum: 6 Intraventricular: 3	Not specified	China	Laterality not reported	Not specified	Although total resection was an independent factor for survival, it could not be achieved in the majority of cases.
Ali 2014 [54]	Retrospective study, 9 patients	9/9 (100%) Frontal: 3 Parietal: 1 Occipital: 1 Temporal:1 Fronto-temporal: 2 Fronto-temporo-parietal: 1	Not specified	USA	R. hemisphere: 7 L. hemisphere: 2	5 female; 4 male.	The article did not establish a relationship between the region of the glioblastoma and clinical implications.
Mohan 2019 [55]	Retrospective study, 65 patients	48/65 (73.85%) Frontal: 11 Temporal: 12 Parietal: 10 Occipital: 3 Two lobes involved: 13	Not specified	USA	Laterality not reported	18 female; 30 men.	The article did not establish a relationship between the region of the glioblastoma and clinical implications.
Mangla 2010 [56]	Retrospective study, 36 patients	36/36 (100%) Fronto-temporal: 22 Others: 14	Not specified	USA	Laterality not reported	12 female; 24 male.	The article did not establish a relationship between the region of the glioblastoma and clinical implications.
Adeberg 2014 [57]	Retrospective study, 100 patients	100/100 (100%) R. hemisphere: 40 L. hemisphere: 47 Bilateral: 13	Not specified	Germany	R. hemisphere: 40 L.hemisphere: 47 Bilateral: 13	41 female; 59 male.	The article did not establish a relationship between the region of the glioblastoma and clinical implications.
wright 2016 [58]	Retrospective study, 10 patients	8/10 (80%) Frontal: 3 Anterior corpus callosum: 1 Corpus callosum: 1 Thalamus: 1 Fronto-parietal: 1 Fronto-temporal: 1	Not specified	USA	R. hemisphere: 4 L. hemisphere: 2 Bilateral: 2	5 female; 5 male.	The article did not establish a relationship between the region of the glioblastoma and clinical implications.
shibahara 2019 [59]	Retrospective study, 87 patients	87/87 (100%) Frontal: 28. Temporal: 30. Parietal: 15. Infratentorial: 2. Others: 12.	Not specified	Japan	Laterality not reported	26 female; 61 male.	The article did not establish a relationship between the region of the glioblastoma and clinical implications.
tykocinski 2012 [60]	Retrospective study, 132 patients	132/132 (100%) Frontal, parietal, occipital: 81 Temporal, insula: 50 Posterior fossa: 1	Not specified	USA	Laterality not reported	61 female; 71 male.	The article did not establish a relationship between the region of the glioblastoma and clinical implications.
Kanas 2017 [61]	Retrospective study, 86 patients	86/86 (100%) Frontal: 25 Temporal: 38 Parietal: 17 Occipital: 6	Not specified	France	R. hemisphere: 47 L. hemisphere: 39	27 female; 59 men.	The article did not establish a relationship between the region of the glioblastoma and clinical implications.
reimer 2017 [62]	Retrospective study, 35 patients	35/35 (100%) Frontal and temporal: 33 Parietal: 2.	Not specified	Germany	Laterality not reported	9 female; 26 male.	The article did not establish a relationship between the region of the glioblastoma and clinical implications.
Jiguet-Jiglaire 2022 [63]	Retrospective study, 38 patients	38/38 (100%) Frontal: 17. Temporal: 15. Corpus callosum: 12. Basal ganglia: 11. several patients presented with more than one location.	Not specified	France	Laterality not reported	27 female; 11 male.	The article did not establish a relationship between the region of the glioblastoma and clinical implications.
Senders 2020 [64]	Retrospective study, 562 patients	562/562 (100%) Frontal: 235. Temporal: 250. Parietal: 175. Occipital: 73. Corpus callosum:: 59. several patients presented with more than one location.	Not specified	USA	R. hemisphere: 302 L. hemisphere: 281; the number of bilateral patients was not specified	Not specified	The article did not establish a relationship between the region of the glioblastoma and clinical implications.
Zhang 2021 [65]	Retrospective study, 60 patients	60/60 (100%) Frontal: 29. Temporal: 13. Parietal: 7. Occipital: 5. Insula, thalamus, others: 6.	Not specified	China	R. hemisphere: 33 L. hemisphere: 27	26 female; 34 male.	The article did not establish a relationship between the region of the glioblastoma and clinical implications.
Liu 2023 [66]	Retrospective study, 60 patients	76/118 (64.41%) R. hemisphere: 35 L. hemisphere: 25 Bilateral: 6 Only 66 were mentioned in terms of laterality, but there were 76 patients with GBM	Not specified	China	R. hemisphere: 35 L. hemisphere: 25 Bilateral: 6;. only 66 were mentioned in terms of laterality, but there were 76 patients with GBM.	Not specified	The article did not establish a relationship between the region of the glioblastoma and clinical implications.
cohen 2000 [67]	Retrospective study, 7 patients	1/7 (14.29%) Occipital	Not specified	Israel	L. hemisphere: 1	1 male	The article did not establish a relationship between the region of the glioblastoma and clinical implications.
nishio 1997 [68]	Retrospective study, 20 patients	4/20 (20%) Thalamus: 4	Not specified	Japan	Laterality not reported	Not specified	The article did not establish a relationship between the region of the glioblastoma and clinical implications.
wang 2015 [69]	Retrospective study, 400 patients	200/400 (50%) Frontal: 109 Temporal: 92 Others: 76	Not specified	China	R. hemisphere: 87 L. hemisphere: 113	71 female; 129 male.	In the GBM cohort, the brain region (Cluster 3) associated with advanced age at tumor diagnosis was mainly located in the bilateral temporal lobe, particularly at the posterior region of the subventricular zone (SVZ). Meanwhile, the brain region associated with younger age at tumor diagnosis was preferentially located in the left inferior frontal region.
simonet-redondo 2012 [70]	Retrospective study, 6 patients	5/6 (83.33%) Frontal: 2 Temporal: 3	Not specified	Spain	R. hemisphere: 37 L. hemisphere: 2	2 female; 3 male.	The article did not establish a relationship between the region of the glioblastoma and clinical implications.
sunwoo 2015 [71]	Retrospective study, 72 patients	20/72 (27.78%) Frontal: 11 Temporal: 7 Parietal: 7 Occipital: 2 Multiple patients presented with more than one location	Not specified	South Korea	Laterality not reported	9 female; 11 male.	The article did not establish a relationship between the region of the glioblastoma and clinical implications.
friese 2000 [72]	Retrospective study, 59 patients	1/59 (1.69%) Corpus callosum: 1	Not specified	Germany	Midline: 1	1 male	The article did not establish a relationship between the region of the glioblastoma and clinical implications.
okamoto 2002 [73]	Retrospective study, 5 patients	2/5 (40%) Parietal: 1 Corpus callosum to right hemisphere: 1	Not specified	Japan	R. hemisphere: 1 L. hemisphere: 1	1 female; 1 male.	The article did not establish a relationship between the region of the glioblastoma and clinical implications.
Stummer 2008 [74]	Prospective study, 243 patients.	221/243 (90.9%) R. hemisphere: 156 L. hemisphere: 87	*p*-value 0.4734 in relation to the difference between hemispheres.	Germany	R. hemisphere: 156 L. hemisphere: 87 These numbers are from the total studied patients, but they were not specifically reported for glioblastoma.	90 female; 153 male.	The article did not establish a relationship between the region of the glioblastoma and clinical implications.
Fudaba 2021 [75]	Prospective study, 35 patients	35/35 (100%) R. hemisphere: 18 L. hemisphere and bilateral: 17	*p*-value: 0.739 in relation to the location in the hemisphere of the tumor. *p*-value: 0.023 in relation to patients who with total or subtotal resection of the tumor showing significantly better progression-free survival.	Japan	R. hemisphere: 18. L. hemisphere and bilateral: 17; the difference between left and bilateral locations was not specified.	14 female; 21 male.	The extension and tumor location on the hemispheric side were not identified as significant predictors of overall survival. However, patients with total or subtotal resection showed significantly better progression-free survival.
Wang 2019 [76]	Prospective study, 109 patients	81/109 (74,3%) Frontal: 13 Parietal: 8 Temporal: 32 Occipital: 3 Insula: 3 Corpus callosum: 6 Basal ganglia: 9 Thalamus: 4 Hippocampus: 3	Not specified	China	Laterality not reported	39 female; 42 male.	Differences in predilection sites were found between GBM and primary central neural system lymphoma.
Mizumoto 2016 [77]	Prospective study, 46 patients	46/46 (100%) Frontal: 23 Temporal: 16 Parietal: 3 Occipital: 4	Not specified	Japan	Laterality not reported	22 female; 24 male.	The article did not establish a relationship between the region of the glioblastoma and clinical implications.
Isoardo 2012 [78]	Prospective study, 19 patients	19/19 (100%) Frontal: 9 Temporal: 5 Parietal: 5	Not specified	Italy	Laterality not reported	6 female; 13 male.	The article did not establish a relationship between the region of the glioblastoma and clinical implications.
Stumpo 2021 [79]	Prospective study, 7 patients	7/7 (100%) Frontal: 4 Temporal: 2 Parietal: 1	Not specified	Switzerland	R. hemisphere: 2 L. hemisphere: 4 Midline: 1	1 female; 6 male.	The article did not establish a relationship between the region of the glioblastoma and clinical implications.
Iliadis 2011 [80]	Prospective study, 65 patients	65/65 (100%) Parietal: 20 Temporal: 20 Frontal: 17 Occipital: 3	Not specified	Greece	R. hemisphere: 29 L. hemisphere: 33 Bilateral: 3	28 female; 37 male.	The article did not establish a relationship between the region of the glioblastoma and clinical implications.
Galldiks 2012 [81]	Prospective study, 25 patients	25/25 (100%) Frontal: 9 Temporal: 7 Parietal: 8 Occipital: 1	Not specified	Germany	Laterality not reported	10 female; 15 male.	The article did not establish a relationship between the region of the glioblastoma and clinical implications.
Henker 2015 [82]	Prospective study, 20 patients	20/20 (100%) Frontal: 6 Temporal: 10 Parietal: 2 Others: 2	Not specified	Germany	R. hemisphere: 11 L. hemisphere: 9	11 female; 9 male.	PTE volume potentially represents infiltration into the tumor area instead of a simple accumulation of water as a side effect of the tumor. (XXXX) may have a benefit in the survival of patients with GBM.
Najafi 2012 [83]	Prospective study, 12 patients	12/12 (100%) Frontal: 4 Temporal: 5 Parietal: 1 Occipital: 1 Multiple: 1	Not specified	Iran	R. hemisphere: 5 L. hemisphere: 6 Multiple: 1	3 female; 9 male.	The article did not establish a relationship between the region of the glioblastoma and clinical implications.
Coburger 2015 [84]	Prospective study, 20 patients	20/20 (100%) Frontal: 10 Temporal: 5 Parietal: 3 Occipital: 2	Not specified	Germany	Laterality not reported	Not specified	The article did not establish a relationship between the region of the glioblastoma and clinical implications.
Hakyemez 2004 [85]	Prospective study, 33 patients	18/33 (55%) Frontal: 4 Temporal: 4 Parietal: 5 Occipital: 3Thalamus: 2	Not specified	Turkey	Laterality not reported	3 female; 15 male.	The article did not establish a relationship between the region of the glioblastoma and clinical implications.
Yu 2017 [86]	Prospective study, 88 patients	43/88 (48%) Cerebral parenchyma: 35 Brainstem: 2 Cerebellar hemisphere: 6	Not specified	China	Laterality not reported	26 female; 62 male.	There was no difference between sexes for GBM and SBM; however, patients with SBM were older than those with GBM.
Laule 2017 [87]	Prospective study, 3 patients	1/3 (33.33%) Frontal: 1	Not specified	Canada	L. hemisphere: 1	1 female	The article did not establish a relationship between the region of the glioblastoma and clinical implications.
Makino 2011 [88]	Prospective study, 21 patients	7/21 (33.33%) Temporal: 3 Frontal: 1 Basal ganglia: 1 Corpus callosum: 1 Thalamus: 1	Not specified	Japan	Laterality not reported	8 female; 13 male.	The article did not establish a relationship between the region of the glioblastoma and clinical implications.
schneider 2005 [89]	Prospective study, 31 patients	31/31 (100%) Parietal: 5 Temporal: 6 Frontal: 7 Fronto-parietal: 7 Fronto-temporal: 2 Parieto-temporal: 2 Parieto-occipital:1 Occipito-temporal: 1	Not specified	Germany	R. hemisphere: 14 L. hemisphere: 17	12 female; 19 male.	The article did not establish a relationship between the region of the glioblastoma and clinical implications.
weber 1999 [90]	Prospective study, 10 patients	10/10 (100%) Frontal: 3 Occipital: 1 Parietal:1 Fronto-parietal: 1 Parieto-occipital: 1 Temporo-parieto-occipital: 2 Fronto-temporal: 1	Not specified	Germany	R. hemisphere: 5 L. hemisphere: 5	3 female; 7 male.	The article did not establish a relationship between the region of the glioblastoma and clinical implications.
Oriuchi 1996 [91]	Prospective study, 20 patients	5/20 (25%) Parietal: 3 Thalamus: 2	Not specified	Japan	R. hemisphere: 2 L. hemisphere: 3	5 male	The article did not establish a relationship between the region of the glioblastoma and clinical implications.
Anzai 1995 [92]	Prospective study, 12 patients	1/12 (8.33%) Frontal: 1	Not specified	USA	L. hemisphere: 1	1 female	The article did not establish a relationship between the region of the glioblastoma and clinical implications.
Todo 2022 [93]	Prospective study, 19 patients	19/19 (100%) Frontal: 11 Temporal: 4 Parietal: 3 Corpus callosum: 1	Not specified	Japan	R. hemisphere: 11 L. hemisphere: 8 Midline: 1	4 female; 15 male.	The article did not establish a relationship between the region of the glioblastoma and clinical implications.
Nakai 2004 [94]	Prospective study, 10 patients	2/10 (20%) Fronto-temporal:1 Temporal: 1	Not specified	Japan	L. hemisphere: 2	1 female; 1 male.	The article did not establish a relationship between the region of the glioblastoma and clinical implications.
Doknic 2020 [95]	Prospective study, 5 patients	1/5 (20%) Temporal: 1	Not specified	Serbia	L. hemisphere: 1 according to the image of the CT presented.	1 male	The article did not establish a relationship between the region of the glioblastoma and clinical implications.
Verburg 2020 [96]	Prospective study, 20 patients	12/20 (60%) Frontal: 3 Parietal: 6 Occipital: 2 Temporal: 1	Not specified	Netherlands	R. hemisphere: 7 L. hemisphere: 5	5 female; 7 male.	The article did not establish a relationship between the region of the glioblastoma and clinical implications.
Nishio 1998 [97]	Prospective study, 11 patients	1/11 (9.09%) Cerebellum and brainstem: 1	Not specified	Japan	Laterality not reported	1 male	The article did not establish a relationship between the region of the glioblastoma and clinical implications.
ballester 2017 [98]	Prospective study, 6 patients	3/6 (50%) Frontal: 1 Temporal: 2	Not specified	USA	R. hemisphere: 3	3 male	The article did not establish a relationship between the region of the glioblastoma and clinical implications.
kim 2022 [99]	Prospective study, 13 patients	8/13 (61.54%) Temporal: 3 Parietal: 1 Occipital: 1 Frontal: 1 Cerebellum: 1 Thalamus, basal ganglia, and midbrain: 1	Not specified	South Korea	R. hemisphere: 5 L. hemisphere: 2	5 female; 3 male.	The article did not establish a relationship between the region of the glioblastoma and clinical implications.
Prasanna 2019 [100]	Prospective study, 138 patients	138/138 (100%) R. hemisphere: 60 L. hemisphere: 78	Not specified	USA	R. hemisphere: 60 L. hemisphere: 78	52 female; 86 male.	MEDH in AAL regions due to the mass effect was associated with survival for right-hemispheric tumors.
Shen 2017 [101]	Case report, 1 patient	1/1 (100%) Cervical spinal cord (C4–C7): 1	Not specified	China	Laterality not reported	1 female	Primary spinal GBM is a clinically rare entity that progresses rapidly with a dismal prognosis and short survival time.
Petzold 2018 [102]	Case report, 1 patient	1/1 (100%) Frontal: 1	Not specified	Germany	L. hemisphere: 1.	1 female	A supposed but not significant association was found between tumors located in the frontal lobe and mood-related symptoms.
Yan 2017 [103]	Case report, 1 patient	1/1 (100%) Conus medullaris: 1	Not specified	China	Laterality not reported	1 male	Spinal GBM located in the conus medullaris is rare.
Faguer 2014 [104]	Case report, 4 patients	4/4 (100%) Temporal: 1 Parietal: 2 Frontal and parietal:1	Not specified	France	R. hemisphere: 3 L. hemisphere: 1	4 male	The article did not establish a relationship between the region of the glioblastoma and clinical implications.
Karthigeyan 2017 [105]	Case report, 1 patient	1/1 (100%) Petroclival: 1	Not specified	India	L. hemisphere: 1	1 female	The article did not establish a relationship between the region of the glioblastoma and clinical implications.
Amini 2006 [106]	Case report, 3 patients	3/3 (100%) Pineal: 3 Posterior ventricular region: 1 one patient had 2 affected areas.	Not specified	USA	Laterality not reported	1 female; 2 male	GBM of the pineal region is extremely rare and is associated with a bad prognosis. Most patients present signs and symptoms of hydrocephalus and Parinaud syndrome, requiring placement of a ventriculoperitoneal shunt or endoscopic biopsy and third ventriculostomy.
Kajitani 2018 [107]	Case report, 3 patients	3/3 (100%) Cerebellum and Pons: 1 Fronto-temporo-parietal and insula: 1 Fronto-parietal: 1	Not specified	Japan	R. hemisphere: 3 L. hemisphere: 0	2 female; 1 male	The article did not establish a relationship between the region of the glioblastoma and clinical implications.
Roemer 2011 [108]	Case report, 1 patient	1/1 (100%) R. hemisphere	Not specified	USA	R. hemisphere: 1	Female	The article did not establish a relationship between the region of the glioblastoma and clinical implications.
Kiang 2021 [109]	Case report, 1 patient	1/1 (100%) Basal ganglia and frontal: 1	Not specified	China	L. hemisphere: 1	Female	The article did not establish a relationship between the region of the glioblastoma and clinical implications.
Boikov 2013 [110]	Case report, 1 patient	1/1 (100%) Thalamus: 1	Not specified	USA	R. hemisphere: 1	Female	The article did not establish a relationship between the region of the glioblastoma and clinical implications.
takahashi 2013 [111]	Case report, 1 patient	1/1 (100%) Frontal: 1	Not specified	Japan	L. hemisphere:1	Male	The article did not establish a relationship between the region of the glioblastoma and clinical implications.
Colombo 2015 [112]	Case report, 1 patient	1/1 (100%) Parietal: 1	Not specified	italy	R. hemisphere: 1	Not specified	The article did not establish a relationship between the region of the glioblastoma and clinical implications.
Nestler 2007 [113]	Case report, 3 patients	3/3 (100%) Cerebellar:1 Parasagittal: 1 Frontal: 1	Not specified	Germany	R. hemisphere: 0 L. hemisphere: 3	1 female; 2 male.	The article did not establish a relationship between the region of the glioblastoma and clinical implications.
Park 2022 [114]	Case report, 1 patient	1/1 (100%) Right posterior temporal and occipital: 1	Not specified	USA	R. hemisphere: 1	1 female	The article did not establish a relationship between the region of the glioblastoma and clinical implications.
Gu 2011 [115]	Case report, 1 patient	1/1 (100%) Fronto-temporal: 1	Not specified	USA	R. hemisphere: 1	1 male	The article did not establish a relationship between the region of the glioblastoma and clinical implications.
soleman 2017 [116]	Case report, 1 patient	1/1 (100%) Frontal: 1	Not specified	Israel	R. hemisphere: 1	1 male	The article did not establish a relationship between the region of the glioblastoma and clinical implications.
Lrhezzioui 2007 [117]	Case report, 1 patient	1/1 (100%) Frontal: 1	Not specified	France	R. hemisphere:1	1 male	The article did not establish a relationship between the region of the glioblastoma and clinical implications.
cohen-gadol 2004 [118]	Case report, 2 patients	2/2 (100%) Precentral gyrus: 1 Temporal: 1	Not specified	USA	R. hemisphere: 2	2 male	The article did not establish a relationship between the region of the glioblastoma and clinical implications.
Lam 2011 [119]	Case report, 2 patients	1/2 (50%) Thalamus and corpus callosum: 1	Not specified	Singapore	L. hemisphere: 1	1 female	The article did not establish a relationship between the region of the glioblastoma and clinical implications.
wu 2002 [120]	Case report, 1 patient	1/1 (100%) Temporal: 1	Not specified	China	L. hemisphere: 1	1 male	The article did not establish a relationship between the region of the glioblastoma and clinical implications.
franco 2000 [121]	Case report, 4 patient	3/4 (75%) Frontal: 1 Right temporo-thalamic and left frontal horn: 1 Frontal and ear of the right lateral ventricle: 1	Not specified	Brazil	R. hemisphere: 2 L. hemisphere: 0 Bilateral: 1	2 female; 1 male.	The article did not establish a relationship between the region of the glioblastoma and clinical implications.
Li 2015 [122]	Case report, 2 patients	2/2 (100%) Temporal: 1 Cerebellum: 1	Not specified	China	R. hemisphere: 1 L. hemisphere: 1	2 female	The article did not establish a relationship between the region of the glioblastoma and clinical implications.
Dilber 2020 [123]	Case report, 2 patients	1/2 (50%) Parieto-temporo-occipital and corpus callosum: 1	Not specified	Turkey	L. hemisphere: 1	1 male	The article did not establish a relationship between the region of the glioblastoma and clinical implications.
ishikawa 2017 [124]	Case report, 15 patients	5/15 (33.33%) Frontal: 1 Temporal: 1 Occipital: 2 Parietal: 1	Not specified	Japan	R. hemisphere: 2 L. hemisphere: 3	3 female; 2 male.	The article did not establish a relationship between the region of the glioblastoma and clinical implications.
Roetzer 2018 [125]	Cadaveric study, 3 patients	3/3 (100%) Temporo-cccipital: 1 Temporal: 1 Pons: 1	Not specified	Austria	R. hemisphere: 2 L. hemisphere: 0 Midline: 1	2 female; 1 male	The article did not establish a relationship between the region of the glioblastoma and clinical implications.
Nguyen 2016 [126]	Cadaveric study, 6 patients	6/6 (100%) Corpus callosum: 2 Corona radiate: 2 Centrum semiovale: 2	Not specified	USA	Laterality not reported	1 female; 5 male	The article did not establish a relationship between the region of the glioblastoma and clinical implications.
Schiff 1998 [127]	Cadaveric study, 3 patients	1/3 (33.33%) Temporo-parietal and basal ganglia: 1	Not specified	USA	L. hemisphere	1 female	The article did not establish a relationship between the region of the glioblastoma and clinical implications.
Ellingson 2011 [128]	Transversal study, 25 patients	25/25 (100%) Frontal: 7 Parietal: 10 Temporal: 5 Occipital: 2	Not specified	USA	Laterality not reported	11 female; 14 male	The article did not establish a relationship between the region of the glioblastoma and clinical implications.
Jallo 1997 [129]	Transversal study, 26 patients	5/26 (19.23%) Frontal: 3 Parietal: 1 Temporal: 1	Not specified	USA	Laterality not reported	1 female; 4 male	The article did not establish a relationship between the region of the glioblastoma and clinical implications.
Maslehaty 2011 [130]	Case report and literature review (1 report/19 articles reviewed)	20/20 (100%) Frontal: 5 Temporal: 5 Parietal: 2 temporo-parietal: 7 fronto-temporal: 2	Not specified	Switzerland	L. hemisphere: 1 (case); literature review not reported	7 female; 13 male	The article did not establish a relationship between the region of the glioblastoma and clinical implications.

**Table 2:** Presence of glioblastoma in cerebral hemispheres.

Author y N	Right Hemisphere	Left Hemisphere	Bilateral
Stummer 221 patients	156	87	-
Drabycz 72 patients	34	38	-
Fudaba 35 patients	18	17	-
Abecassis 31 patients	20	30 (patients with gliomas)	-
Stark 7 patients	-	1	-
Petzold 1 patients	-	1	-
Chen 15 patients	4	9	-
Stumpo 7 patients	2	4	1
Awad 330 patients	168	139	23
Cui 77 patients	-	-	36
Wach 198 patients	111	87	-
Steidl 65 patients	36	28	1
Ideguchi 5 patients	3	2	-
Wang 19 patients	6	6	1
Faguer 4 patients	3	1	-
Muller 275 patients	141	134	-
Iliadis 65 patients	29	33	3
Karthigeyan 1 patient	-	1	-
Smedley 304 patients	162	149	-
Han 92 patients	39	39	14.
Henker 20 patients	11	9	-
Roetzer 3 patients	2	-	-
Fan 133 patients	56	77	-
Najafi 12 patients	5	6	-
Wang 153 patients	73	80	-
Smets 24 patients	15	9	-
Kajitani 3 patients	3	-	-
Roemer 1 patient	1	-	
Kiang 1 patient	-	1	-
Boikov 1 patient	1	-	-
Sugimoto 4 patients	-	4	-
Laule 1 patient	-	1	-
Olar 4 patients	1	3	-
Takahashi 1 patient	-	1	-
Ali 9 patients	7	2	-
Colombo 1 patient	1	-	-
Nestler 3 patients	-	3	-
Mohan 48 patients	-	-	-
Schiff 1 patient	-	1	-
Maslehaty 19 patients	-	1	-
Park 1 patient	1	-	-
Adaberg 100 patients	40	47	13
Gu 1 patient	1	-	-
Wright 8 patients	4	2	2
Schneider 31 patients	14	17	-
Soleman 1 patient	1	-	-
Irhezzioui 1 patient	1	-	-
Cohen-gadol 2 patients	2	-	-
Iam 1 patient	-	1	-
Weber 10 patients	5	5	-
Wu 1 patient	-	1	-
Franco 3 patients	2	-	1
Oriuchi 5 patients	2	3	-
Kanas 86 patients	47	39	-
Anzai 1 patient	-	1	-
Li 2 patients	1	1	-
Senders 562 patients	302	281	-
Todo 19 patients	11	8	1
Nakai 2 patients	-	2	-
Zhang 60 patients	33	27	-
Liu 76 patients	25	35	6
Doknic 1 patient	-	1	-
Dilber 1 patient	-	1	-
Cohen 1 patient	-	1	-
Verburg 20 patients	7	5	-
Wang 200 patients	87	113	-
Simonet Redondo 5 patients	3	2	-
Friese 1 patient	-	-	1 (callosum body)
Okamoto 2 patients	1	1	1 (callosum body)
Nishio 1 patient	-	-	1 (cerebellum)
Ballester 3 patients	3	-	-
Ishikawa 5 patients	2	3	-
Kim 8 patients	5	2	1 (posterior fossa)
Prasanna 138 patients	60	78	-

**Table 3:** Mean and standard deviation in the presence of glioblastoma per cerebral hemisphere.

Hemisphere	Right Hemisphere	Left Hemisphere
Median	33.36	34.70
Standard deviation	58.00	65.07

**Table 4:** Presence of glioblastoma in cerebral lobes and brain regions.

Author and Number of Patients	Frontal Lobe	Parietal Lobe	Temporal Lobe	Occipital Lobe	Insula	Diencephalom	Brain Stem	Cerebellum	Other Structures
Hashiguchi 51 patients	15	8	16	3	-	-	-	2	7
Drabycz 72 patients	19	17	32	2	-	-	-	-	2
Chung Ko 104 patients	38	25	27	3	-	5	1	-	5
Abecassis 31 patients	17	8	24	-	-	-	1	-	-
Wang 81 patients	13	8	32	3	3	7	-	-	15
Shen 1 patients	-	-	-	-	-	-	-	-	1
Mizumoto 46 patients	23	3	16	4	-	-	-	-	-
Isoardo 19 patients	9	5	5	-	-	-	-	-	-
Ferreira 67 patients	33	14	14	6	-	-	1	-	4
Stark 7 patients	-	-	-	-	-	-	2	4	1
Liu 107 patients	-	-	-	-	-	-	-	-	153
Petzold 1 patient	1	-	-	-	-	-	-	-	-
Chen 15 patients	5	1	4	3	-	-	1	1	2
Stumpo 7 patients	4	1	2	-	-	-	-	-	-
Jaskólski 26 patients	11	6	7	2	-	-	-	-	-
Kim 83 patients	-	-	29	-	-	-	-	-	54
Hatakeyama 55 patients	-	-	-	-	-	-	-	1	54
Yamashita 112 patients	36	25	21	3	7	-	-	-	20
Kuroiwa 9 patients	-	-	-	-	-	-	4	9	-
Awad 330 patients	132	99	139	50	-	-	4	3	200
Quan 110 patients	42	-	-	-	-	-	-	-	68
Onuma 33 patients	17	-	-	-	-	-	-	-	16
Cui 77 patients	44	11	-	11	22	-	-	-	77
Steidl 65 patients	16	15	27	12	1	2	-	-	1
Park 108 patients	45	-	-	-	-	-	-	-	63
Yan 1 patient	-	-	-	-	-	-	-	-	1
Ideguchi 5 patients	1	1	1	1	-	-	-	-	1
Wang 19 patients	9	2	3	1	-	-	1	-	6
Faguer 4 patients	1	3	1	-	-	-	-	-	-
Thomas 21 patients	6	-	1	-	4	-	-	-	10
Iliadis 65 patients	17	20	20	3	-	-	-	-	8
Galldiks 25 patients	9	8	7	1	-	-	-	-	-
Koike 11 patients	-	-	-	-	-	-	-	-	11
Xing 75 patients	32	12	13	5	1	-	-	-	12
Karthigeyan 1 patient	-	-	-	-	-	-	-	-	1
Smedley 304 patients	114	76	91	18	-	10	-	1	-
Michelini 42 patients	-	-	-	-	-	-	-	-	42
Henker 20 patients	6	2	10	-	-	-	-	-	2
Mathew 47 patients	-	-	-	-	-	-	-	-	56
Jiang 10 patients	-	1	-	1	-	-	-	-	9
Roetzer 3 patients	-	-	2	1	-	-	1	-	-
Yu li 406 patients	182	-	224	-	-	-	-	-	-
Utsuki 37 patients 5 patients	17	8	8	9	-	-	-	-	-
Hart 5 patients	1	3	-	1	-	-	-	-	-
Najafi 12 patients	4	1	5	1	-	-	-	-	1
Smets 24 patients	5	12	8	6	-	-	-	-	-
Amini 3 patients	-	-	-	-	-	3	-	-	1
Kajitani 3 patients	2	2	1	-	1	-	1	1	-
Coburger 20 patients	10	3	5	2	-	-	-	-	-
Kiang 1 patient	1	-	-	-	-	-	-	-	1
Boikov 1 patient	-	-	-	-	-	1	-	-	-
Hakyemez 18 patients	4	5	4	3	-	2	-	-	-
Eoli 86 patients	36	-	35	-	-	-	-	-	15
Yu 43 patients	-	-	-	-	-	-	2	6	35
Sugimoto 4 patients	3	-	1	-	-	-	-	1	1
Laule 1 patient	1	-	-	-	-	-	-	-	-
Makino 7 patients	1	-	3	-	-	1	-	-	2
Seidel 122 patients	70	38	62	38					74
Cho 60 patients	33	21	34	4	8	5	-	-	24
Olar 4 patients	1	2	2		1				1
Takahashi 1 patient	1	-	-	-	-	-	-	-	-
Woo 147 patients	46	34	47	5	3	-	-	6	6
Nguyen 6 patients	-	-	-	-	-	-	-	-	6
Ali 9 patients	6	2	4	1	-	-	-	-	-
Colombo 1 patient	-	1	-	-	-	-	-	-	-
Nestler 3 patients	1	-	-	-	-	-	-	1	1
Mohan 48 patients	11	10	12	3	-	-	-	-	-
Mangla 36 patients	22	-	22	-	-	-	-	-	14
Schiff 1 patient	-	1	1	-	-	-	-	-	1
Maslehaty 19 patients	7	9	14	-	-	-	-	-	-
Park 1 patient	-	-	1	1	-	-	-	-	-
Ellingson 25 patients	7	10	5	2	-	-	-	-	-
Gu 1 patient	1	-	1	-	-	-	-	-	-
Wright 8 patients	5	1	1	-	-	1	-	-	2
Schneider 31 patients	16	15	11	2	-	-	-	-	-
Soleman 1 patient	1	-	-	-	-	-	-	-	-
Irhezzioui 1 patient	1	-	-	-	-	-	-	-	-
Cohen-gadol 2 patients	1	-	1	-	-	-	-	-	-
Iam 1 patient	-	-	-	-	-	1	-	-	1
Weber 10 patients	5	4	3	3	-	-	-	-	-
Wu 1 patient	-	-	1	-	-	-	-	-	-
Franco 3 patients	3	-	1	-	-	1	-	-	1
Oriuchi 5 patients	-	3	-	-	-	2	-	-	-
Shibahara 87 patients	28	15	30	-	-	-	-	-	14
Tykocinski 132 patients	81	81	50	81	50	-	-	-	1
Kanas 86 patients	25	17	38	6	-	-	-	-	-
Anzai 1 patient	1	-	-	-	-	-	-	-	-
Reimer 35 patients	33	2	33	-	-	-	-	-	-
Li 2 patients	-	-	1	-	-	-	-	1	-
Jiguet-Jiglaire 38 patients	17	-	15	-	-	-	-	-	23
Senders 562 patients	235	175	250	73	-	-	-	-	59
Todo 19 patients	11	3	4	-	-	-	-	-	1
Nakai 2 patients	1	-	2	-	-	-	-	-	-
Zhang 60 patients	29	7	13	5	-	-	-	-	6
Doknic 1 patient	-	-	1	-	-	-	-	-	-
Jallo 5 patients	3	1	1	-	-	-	-	-	-
Dilber 1 patient	-	1	1	1	-	-	-	-	2
Cohen 1 patient	-	-	-	1	-		-	-	-
Nishio 4 patients	-	-	-	-	-	4	-	-	-
Verburg 12 patients	3	6	1	2	-	-	-	-	-
Wang 200 patients	109	-	92	-	-	-	-	-	76
Simonet Redondo 5 patients	2	-	3	-	-	-	-	-	-
Sunwoo 20 patients	11	7	7	2	-	-	-	-	-
Friese 1 patient	-	-	-	-	-	-	-	-	1
Okamoto 2 patients	-	1	-	-	-	-	-	-	1
Nishio 1 patient	-	-	-	-	-	-	1	1	-
Ballester 3 patients	1	-	2	-	-	-	-	-	-
Ishikawa 5 patients	1	1	1	2	-	-	-	-	-
Kim 8 patients	1	1	3	1	-	1	1	-	2
